# Antitumor effect of IL-12 gene-modified bone marrow mesenchymal stem cells combined with Fuzheng Yiliu decoction in an in vivo glioma nude mouse model

**DOI:** 10.1186/s12967-021-02809-2

**Published:** 2021-04-07

**Authors:** Jianjun Wu, Shoupin Xie, Hailong Li, Yanxia Zhang, Jia Yue, Chunlu Yan, Kai Liu, Yongqi Liu, Rui Xu, Guisen Zheng

**Affiliations:** 1 School of Public Health, Gansu University of Chinese Medicine, Lanzhou, 730000 Gansu China; 2 Key Laboratory of Dunhuang Medicine and Transformation at Provincial and Ministerial Level, Lanzhou, 730000 Gansu China; 3 Provincial Key Laboratory of Chinese Medicine Prevention and Control of Chronic Diseases, Lanzhou, 730000 Gansu China; 4 Department of Neurology, The First People’s Hospital of Lanzhou City, Lanzhou, 730050 China; 5 School of Basic Medicine, Gansu University of Chinese Medicine, Lanzhou, 730000 Gansu China; 6 School of Integrated Chinese and Western Medicine, Gansu University of Chinese Medicine, Lanzhou, 730000 Gansu China

**Keywords:** Bone marrow mesenchymal stem cell, Glioma, IL-12, Fuzheng Yiliu decoction

## Abstract

**Background:**

Glioma is a complex cancer with a high morbidity and high mortality. Bone marrow mesenchymal stem cells (BMSCs) have shown promise as an excellent cell/drug delivery vehicle for gene-targeted therapy; however, maintaining genetic stability and biological activity remains difficult. Furthermore, whether BMSCs support or inhibit tumor growth remains debated. This study investigated whether a traditional Chinese medicine fomular, Fuzheng Yiliu decoction (FYD) had a synergistic antitumor effect with IL-12 gene-modified BMSCs in glioma-bearing nude mice

**Methods:**

The lentivirus-mediated IL-12 gene was transfected into primarily cultured BMSCs. A total of 72 BALB/c nude mice were used to establish xenograft models with glioma U251 cells and were divided into groups (n = 12) including blank control group, nude mouse model group (model group), lentiviral transfection of BMSC group with no gene loading (BMSC group), IL-12 lentivirus-transfected BMSC group (IL-12 + BMSC group), FYD treatment group (FYD group), and FYD treatment in IL-12 lentivirus-transfected BMSC group (FYD + IL-12 + BMSC group).. After treatment for 14 days, all mice were sacrificed to collect tumor tissue and serum for more detection, such as distribution of BMSCs, cell apoptosis in xenograft tumors, serum IL-12 and INF-γ levels, mouse weight and tumor volume were measured

**Results:**

There were significantly more apoptotic cells in tumor tissue in IL-12 gene transfected group, FYD treatment group and FYD combining with IL-12 gene transfected group than that in the model group (*P* < 0.05). The FYD + IL-12 + BMSC group showed significantly higher Bax and lower Bcl-2 expression (*P* < 0.05), and serum IL-12 and INF-γ levels (*P* < 0.05) were higher than that in all other groups. After the intervention, this group also showed a strong inhibitory effect against tumor growth (*P* < 0.05)

**Conclusions:**

This study suggested FYD treatment combined with IL-12 gene-modified BMSCs shows synergistic antitumor effect in glioma-bearing nude mice.

## Introduction

Glioma accounts for 80% of central nervous system tumors in adults. These lethal brain tumors are aggressive, malignant, and essentially incurable [1], contributing to a major economic burden. As such, new and effective treatments for gliomas are required. Bone marrow mesenchymal stem cells (BMSCs) may represent a potential solution, as they can be characterized by multi-directional differentiation [2], are easily replicated [3], express tumor tropism [4], have low immunogenicity [5], can undergo EasyGene modification [6], and exhibit immune regulation [7]. Therefore, BMSCs could be used as an excellent cell/drug delivery vehicle in cell-based targeted therapies [3], providing an alternative therapeutic approach to immunotherapy, suicide protein therapy, and anti-angiogenesis therapy for treating gliomas [3, 8]. However, for use as a carrier, further research is needed to investigate the effects of BMSCs on tumor growth [6, 9]. Some investigators have found that BMSCs can increase the invasiveness of tumors [10], while others have reported an antitumor effect [11].

BMSCs secrete various modulatory factors in vitro, which contribute to their complex effects on immune suppression [12]. Shirjang et al. [13] recently showed that toll-like receptors (TLRs) were critical in regulating the effect of BMSCs. TLRs are mainly found on the surface of antigen-presenting cells (e.g., dendritic cells) and send a warning signal to the body upon infection. Both TLR2 and TLR4 activate dendritic cells and stimulate the production of various cytokines and chemical activators; for instance, TLR4 mainly stimulates p70, interferon (IFN)-*γ*-induced protein (IP-10), and IFN-β, whereas TLR2 stimulates interleukin (IL)-8 and IL-23 expression. These soluble cytokines can induce T helper (Th) cell activation and function as an immune monitor. In particular, IL-12 can stimulate T cells to produce IFN to effectively promote the differentiation of Th cells into Th1 cells, which then exert an immune function. IL-12 can also stimulate T cells to produce IFN-γ, and BMSCs can increase TLR3 and TLR4 expression after being primed with IFN-γ, effectively promoting antigenic cell activation and causing antitumor immunity [6, 7, 14]. Studies have confirmed that IL-12 or INF gene-modified BMSCs are ideal gene delivery systems for antitumor gene therapy that stably express transfected proteins to exert potent antitumor effects [6, 15]. Thus, gene modification might improve the tumorigenicity of BMSCs in potential antitumor treatments, and many studies have demonstrated methods for maintaining the genetic stability and biological activity of BMSCs [16–18].

Traditional Chinese Medicine (TCM) offers effective treatments to improve immune function, suppress tumor growth, and alleviate the adverse effects of radiotherapy and chemotherapy [19]. Decoctions derived from specific TCMs have demonstrated both attenuation and efficacy in reversing the tumorigenicity of BMSCs, contributing to the treatment of disease [20–23]. The Fuzheng Yiliu decoction (FYD) is a medical prescription comprising red *stilbene*, *Angelica sinensis*, *rhizoma zedoariae*, and *mutouhui* at an herbal dosage ratio of 3:1:3:1. FYD is used as an antitumor medication and has been shown to reverse the tumorigenicity of BMSCs in the tumor microenvironment in vitro [24–27]. In the present study, we evaluated the synergistic antitumor effect of FYD and IL-12 gene-modified BMSCs using a glioma-bearing BALB/c nude mouse model.

## Methods

### Human glioma U251 cell line culture

U251 cells were purchased from the Chinese Academy of Sciences cell bank (Shanghai, China). Cells were incubated at 37 °C, 5% CO_2_, and 100% humidity in Dulbecco’s modified Eagle medium (DMEM; Gibco, USA), and medium was changed once every 1–3 days.

### Primary culture, isolation, and identification of BMSCs

Eight-week-old specific pathogen-free BALB/c mice were anesthetized before undergoing cervical vertebrae dislocation. Their femurs and tibias were then separated under aseptic conditions, and osteophytes were removed. The bone marrow was washed with DMEM until the marrow cavity became white. The cells were aspirated into a centrifuge tube and centrifuged at 1000 rpm for 5 min, after which the supernatant was discarded. Next, the cells were flushed with phosphate-buffered saline (PBS) and supplemented with 15% fetal bovine serum. The medium was discarded and washed to remove any unattached cells after 24 h. New medium was added, and culturing was continued for 3 days, after which a complete medium change was performed again. On day 10, cell growth and confluence generally exceeded 70%. The cell surface molecular markers CD45, CD90 were detected by two-color flow cytometry.

### Xenograft models and grouping of BALB/c nude mice

Adult female BALB/c athymic mice (weighing 15–20 g; aged 6–7 weeks old) were obtained from Beijing Huakang Biotechnology Co., Ltd. The animals were housed in a facility that received 12 h of light per day and were fed ad libitum. The animals were maintained according to the Guide for the Care and Use of Laboratory Animals. During the experimental process, the procedures of feeding, sacrificing or dissecting of the tumors were performed in strict accordance with the international ethical guidelines and the National Institutes of Health Guide for the Care and Use of Laboratory Animals. The study protocol was approved by the Animal Experimental Ethical Inspection of the Gansu University of Chinese Medicine. Every effort was made to avoid unnecessary suffering of the animals.

A number of 12 mice were randomly selected as the blank group and the remaining mice were used to establish the model. A xenograft tumor model was established using glioma cells at a logarithmic growth phase. A 150-μL cell suspension was prepared. The final cell concentration was 10^7^ cells/mL. The 150-µL suspension was subcutaneously injected into the auxiliary region of the mice. The animals were monitored daily for changes in weight, side effects, or sickness post-inoculation. Successful establishment of the mouse model included mice with circular nodules under their skin after 10 days. Finally, the rest of the mice were randomly divided into five groups. At the day after the last treatment, A total of 60 mice were anaesthetized with chloral hydrate with 350 mg/kg injected intraperitoneally, and picked off the eyeballs to collect blood samples for determination of serum IFN-γand IL-12 concentrations. Since then, the number of 15 anaesthetized mice (3 mice in each group) were selected randomly and sacrificed by cervical dislocation method to collect the tumor tissues of all groups for volume and weight measurement, pathological experiment and immunohistochemical detection. Once the mice were then sacrificed, the tumors were dissected and photographed. All mice undertaken anesthesia would accept the isolation of blood and tumor tissues samples in turn without feelings of pain of in a period of planned time. The humane endpoint of mice is judged by massive blood loss and severe anemia, or subcutaneous xenograft tumor reaches to or near 20 mm. Next, all animals which still remained in a state without consciousness would be sacrificed by cervical dislocation treatment till death of all animals was confirmed by the signs including no spontaneous breathing for 2–3 min, no blink reflex existed, and body rigor mortis appeared finally. All operation including anesthesia, injection, sacrifice in animal experiments were performed in strict accordance with the international ethical guidelines and the National Institutes of Health Guide [28] and the AVMA Guidelines for the Euthanasia of Animals, 2013 Edition [29] for the Care and Use of Laboratory Animals so as to guarantee that the end point of mice is humane. The experimental protocol was approved by the Institutional Animal Care and Use Committee of the Gansu University of Chinese Medicine.

### Experimental groups and treatment administration

Mice were randomly divided into six groups (n = 12 per group): blank control group nude mouse model group (model group), lentiviral transfection of BMSC group with no gene loading (BMSC group), IL-12 lentivirus-transfected BMSC group (IL-12 + BMSC group), FYD treatment group (FYD group), and FYD treatment in IL-12 lentivirus-transfected BMSC group (FYD + IL-12 + BMSC group). Prior treatment, the BMSCs were transduced with lentivirus vectors and screened by puromycin to select stably transfected cells. A solution of sample in PBS (10^6^, 200 μL) was injected into the tail vein of mice in the BMSC, IL-12 + BMSC, and FYD + IL-12 + BMSC groups; the model and FYD groups were injected with normal saline. In addition, FYD extract was administered to the FYD and FYD + IL-12 + BMSC groups for 14 days via intragastric administration (0.6 FYD g/20 g body weight, once per day, continuous administration); the model, BMSC, and IL-12 + BMSC groups received an equal volume of distilled water via intragastric administration.

### FYD preparation and quality control

In TCM, FYD is made up of *red stilbene*: *Angelica sinensis*: *rhizoma zedoariae*: *mutouhui* with a ratio of 3:1:3:1 (Table 1). Hong Qi (No. 160627, *Hedysarum polybotrys* Hand.-Mazz.), Dang Gui (No. 160713, *Angelica sinensis* (Oliv.) Diels), Mu Tou Hui (No.160503, *Patrinia scabra* Bunge), and E Zhu (No. 160525, *Curcuma aeruginosa* Roxb.) were purchased from Gansu Fuxing Hou Chinese Medicine Pieces Pharmaceutical Co., Ltd. (Lanzhou City, Gansu Province, China) and carefully authenticated by a professor (Professor Jing Shao) at the School of Pharmacy, Gansu University of Chinese Medicine. The reference standard herbs (red stilbene, *Angelica sinensis*, mutouhui, and rhizoma zedoariae) were purchased from the National Institutes for Food and Drug Control (Beijing, China).

**Table 1:** The information of Chinese medicines in FYD-derived herb

Chinese name	Latin name	Family	Place of origin (province)	Used part	Major effective compound in modern pharmacology study
Hong Qi	*Hedysarumpolybotrys*	*Leguminosae*	Gansu	Rhizome	ononinLigustilide, butylphthalide,
Dang Gui	*Angelica sinensis*	*Umbelliferae*	Gansu	Rhizome	Soya saponin II methyl ester, 4 hydroxy 3 methoxycinnamic acid, Angelica Polysaccharide,
Mu Tou Hui	*Patriniascabra*	*Valerianaceae*	Gansu	Rhizome	Patrinene, isovaleric acid, Triterpenoid saponins
E Zhu	*Curcuma aeruginosa*	*Zingiberaceae*	Guangxi	Rhizome	Curzerenone

The herbs were dried before decoction, mixed to the indicated proportion, and soaked with distilled water for 40 min at room temperature, after which the mixture was decocted three times for 30 min. Gauze was used to filter and remove the dregs. The filtrates were mixed and condensed at 0.09 MPa and 60 °C to a final concentration of 1 g/mL (weight of original herbs/volume of solution) and stored at 4℃.

### IL-12 lentivirus vector construction and transfection

#### Vector design and plasmid constructs

Purification of the target gene by PCR was performed using a plasmid cloning template containing the target gene. The primers were synthesized by Shanghai Jima Pharmaceutical Technology Co., Ltd. (Shanghai, China) and the IL-12 overexpression sequence was amplified by PCR. To subclone the vector, we added the upstream and downstream primers of the target gene to the homologous sequences of NotI and NsiI on the LV5 vector, respectively. The primer sequences for IL-12 were as follows: 5′-AGGGTTCCAAGCTTAAGCGGCCGCGCCACCATGTGGCCCCCTGGGTCAGCCTCCCAG-3′ and 3′-GATCCATCCCTAGGTAGATGCATTTAGGAAGCATTCAGATAGCTC -3′. After completion of the PCR reaction, the IL-12 gene fragment was recovered by agarose gel electrophoresis and gel-cutting.

#### Virus production, gene transduction, and purification

The IL-12 gene fragment was amplified and cloned into a linearized LV5 vector using the ClonExpress® Entry One Step Cloning Kit (TaKaRa, Japan), and the recombinant clone products were transformed into competent cells and that had been previously prepared. The cultured bacterial solution was extracted with a Plasmid Miniprep Kit (Promega, USA), and the extracted plasmid was subjected to double enzyme digestion and identification. Plasmid extraction was carried out to obtain a sufficient amount of the recombinant plasmid. Virus packaging was conducted using a four-plasmid system comprising LV5, PG-p1-VSVG, PG-P2-REV, and PG-P3-RRE. Of these plasmids, LV5 could express green fluorescent protein. All four plasmid vectors were extracted using a high-purity endotoxin-free kit (Biosciences, Americian) and co-transfected into 293T cells. The cell supernatant rich in lentiviral particles was collected and concentrated.

#### Transfection of IL-12-loaded lentivirus into BMSCs

Performed preliminary experiments of lentivirus infection in the target cells. A given volume of virus solution at a multiplicity of infection (MOI) of 10 was transferred to the target cells and control group, and incubated overnight under 5% CO_2_ and 37 °C. We used puromycin to select stably transfected cells. Fluorescence was observed under an inverted fluorescence microscope (Olympus, Japan) to estimate the efficiency of cellular lentiviral infection.

### Verification of IL-12 over expression in BMSCs by quantitative PCR

RNA was reverse transcribed into cDNA using random primers for IL-12, and then specific primers (Weisten Biomedical Technology Co., Ltd., Chongqing, China) and SYBR green I fluorescent dyes (Weisten Biomedical Technology Co., Ltd.) were designed for real-time PCR (Weisten Biomedical Technology Co., Ltd.). The random primer sequences for IL-12 were as follows: 5′-CAGCACTTCAGAATCACAACCA-3′ and 5′-TCATTTTCACTCTGTA AGGGTCTG-3′. The primer sequences for *β*-actin were as follows: 5′-GAGACCTTCAACA CCCCAGC-3′ and 5′-ATGTCACGCACGATTTCCC-3′.

### Distribution of IL-12-transfected BMSCs in mouse tumors

The tumor tissues of all groups were embedded with optimal cutting temperature compound (Sakura Finetek USA, Torrance CA, USA) and stored at − 80 °C until analysis. After sectioning, the fluorescence distribution of IL-12-transfected BMSCs in mice was observed using a laser confocal system (Leica, Germay) with immunofluorescence staining at room temperature.

### Detection of apoptotic tumor cells in mice with TUNEL staining

Mouse tumor tissue was washed with PBS, fixed with 4% paraformaldehyde, and sequentially dehydrated in 75%, 85%, 95%, and 100% ethanol. The tissue was treated with a clearing agent and embedded with wax. Tissue slices were cut to a 5-μm thickness and placed flat on slides. After baking, dewaxing, and rehydration, the cells were stained according to the TUNEL apoptosis detection kit. The number of positive cells for apoptosis/necrosis and the total number of cells were assessed from a random area in each group. The relative proportion of positive cells (positive cells/total cell number) was calculated.

### ELISA detection

The contents of IL-12 and INF-γ in samples were assessed using Human IL-12/IFN-gamma ELISA (Shanghai Lianke Biological Company, China) in microplates according to the manufacturer’s guidelines. During incubation, the IL-12 and INF-γ in the samples combined with the solid-phase antibody. After washing and removing unbound material, the biotinylated detection antibody was added and incubated. Unbound biotinylated antibody was removed by washing, and horseradish peroxidase-labeled streptavidin was added. After washing, the chromogenic substrate TMB was added for color development. The depth of the color reaction was proportional to the concentration of IL-12/INF-γ in the sample. The addition of stop solution terminated the reaction, and the absorbance was measured at a wavelength of 450 nm (reference wavelength: 570 nm).

### Tumor volume measurement

The length and short diameter of tumors in nude mice were measured with Vernier calipers every 2 days. The volume and average volume of the tumors were calculated according to the formula [tumor volume = (*π*/6) × *a* × *b*^2^)] using Microsoft Excel (Microsoft Corp., Redmond, CA, USA). The growth curve of each tumor was drawn according to the volume, where in *a* is the longer and *b* is the shorter axis. The change in weight of each mouse was also recorded.

### Immunohistochemistry assay

Bax and Bcl-2 expression in the tumor tissues of mice were assessed by immunohistochemistry method. Tumor tissue was cut into 4-μm sections. After dewaxing, rehydrating, and washing, tissues were prepared according to standard immunohistochemistry procedures. Finally, the sections were counterstained with hematoxylin and examined under a light microscope (Olympus, Japan).

Three tissue sections from each group of tumor tissue were randomly selected for indexing using five randomly selected fields of view. Signals were recorded using a chemiluminescence imaging analysis system (Chemidoc MP Imaging System; Bio-Rad, Hercules, CA, USA), and the optical density was determined and averaged.

### Statistical analysis

The quantitative data are presented as the mean ± SD. The statistical inference between three or more than groups were calculated using one-way analysis of variance (ANOVA) was used for more than two group testing. The *q* test was used to compare the results between the two groups. A P value of 0.05 was used as the threshold to indicate significance.

In addition, repeated-measures ANOVA was also conducted to test the significance of tumor volume and weight of different groups, firstly, the sphericity test was used to compare the parameter time among different time point or treatment factor among different groups, or the interaction between time and treatment. Once the sphericity test showed that P value < 0.01, the Greenhouse–Geisser model determined that the main effect difference was statistically significant at different time points. Next, Tukey’s test method was used to test the significant differences between any two groups. A P value of 0.05 was used as the threshold to indicate significance.

## Results

### Primary culture, isolation, and identification of BMSCs

**Fig. 1:**
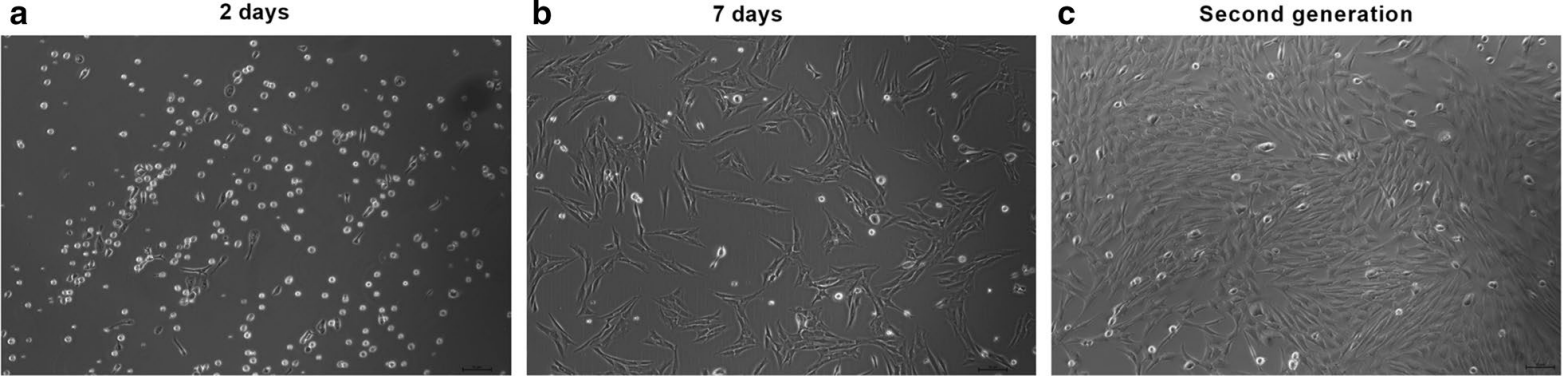
Cell morphology of primary cultured BMSCs at different time points (magnification, × 400). BMSCs were cultured in vitro and adherent growth (primary culture) was observed over a period of 24 h. The cells slowly became round, fusiform and triangular in appearance from 2 days and/or 7 days compared with those from the second generation. Thereafter, the cells proliferated substantially and several spindle-shaped cells appeared. BMSCs, bone marrow mesenchymal stem cells

BMSCs were cultured in vitro and adherent growth (primary culture) occurred over a period of approximately 24 h. The cells slowly became round, fusiform and triangular in appearance. Subsequently, the cells proliferated substantially and several spindle-shaped morphologies appeared (Fig. 1a–c). The results of the flow cytometry analysis indicated that CD90 expression was overexpressed, whereas CD45 expression was downregulated (Table 2).

**Table 2:** Test Results of BMSCs Surface Molecular Marker (CD45, CD90)

Surface marker	F0-2	F0-3	F0-4	F0-5	F0-6	F0-7	F0-8	F0-9	F1-7	F2-7	F3-7
CD45 (%)	65.3	68.4	0.1	1.9	0.3	0.8	0.0	0.0	0.0	0.0	0.0
CD90 (%)	5.8	3.5	6.9	12.0	22.8	61.8	83.8	89.0	94.0	94.9	97.0

### Detection of IL-12 mRNA expression levels in BMSCs by RT-qPCR

**Fig. 2:**
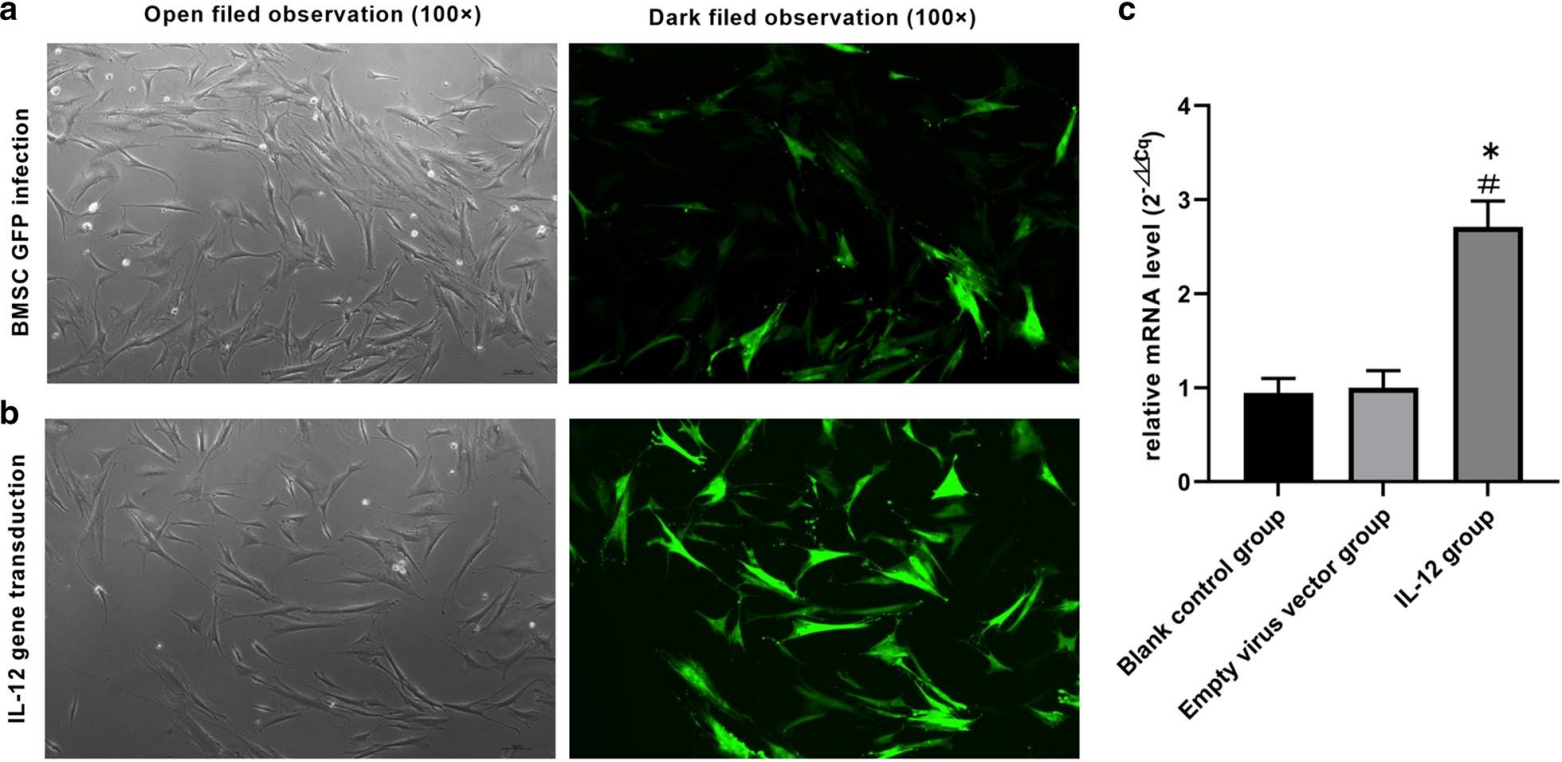
Fluorescent distribution characteristics of BMSCs transfected with IL-12 in nude mice. The relative mRNA expression levels of IL-12 in BMSCs were detected and measured following transfection of the IL-12 gene; the IL-12 mRNA expression levels of the IL-12 + BMSC group were significantly higher than those of the blank control group and BMSC group, as shown in **a**–**c**. (IL-12: F = 351.261, P < 0.001): n = 3, ^#^P < 0.05: Blank control group vs. IL-12 group; ^*^P < 0.05: Empty viral vector group vs*.* the IL-12 group. BMSCs, bone marrow mesenchymal stem cells; IL-12 + BMSC, IL12-lentiviral-transfected BMSC; BMSC group, empty lentiviral vector-transfected BMSC group

The relative mRNA expression levels of IL-12 were detected in BMSCs following transfection of the IL-12 gene. The data indicated that IL-12 expression was significantly lower in the empty lentiviral vector-transfected BMSC group (BMSC group) compared with that noted in the IL-12 lentiviral-transfected BMSC group (IL-12 + BMSC group) (P < 0.05) (Fig. 2a–c).

### Fluorescence distribution of IL-12-transfected BMSCs in mice by confocal microscopy

**Fig. 3:**
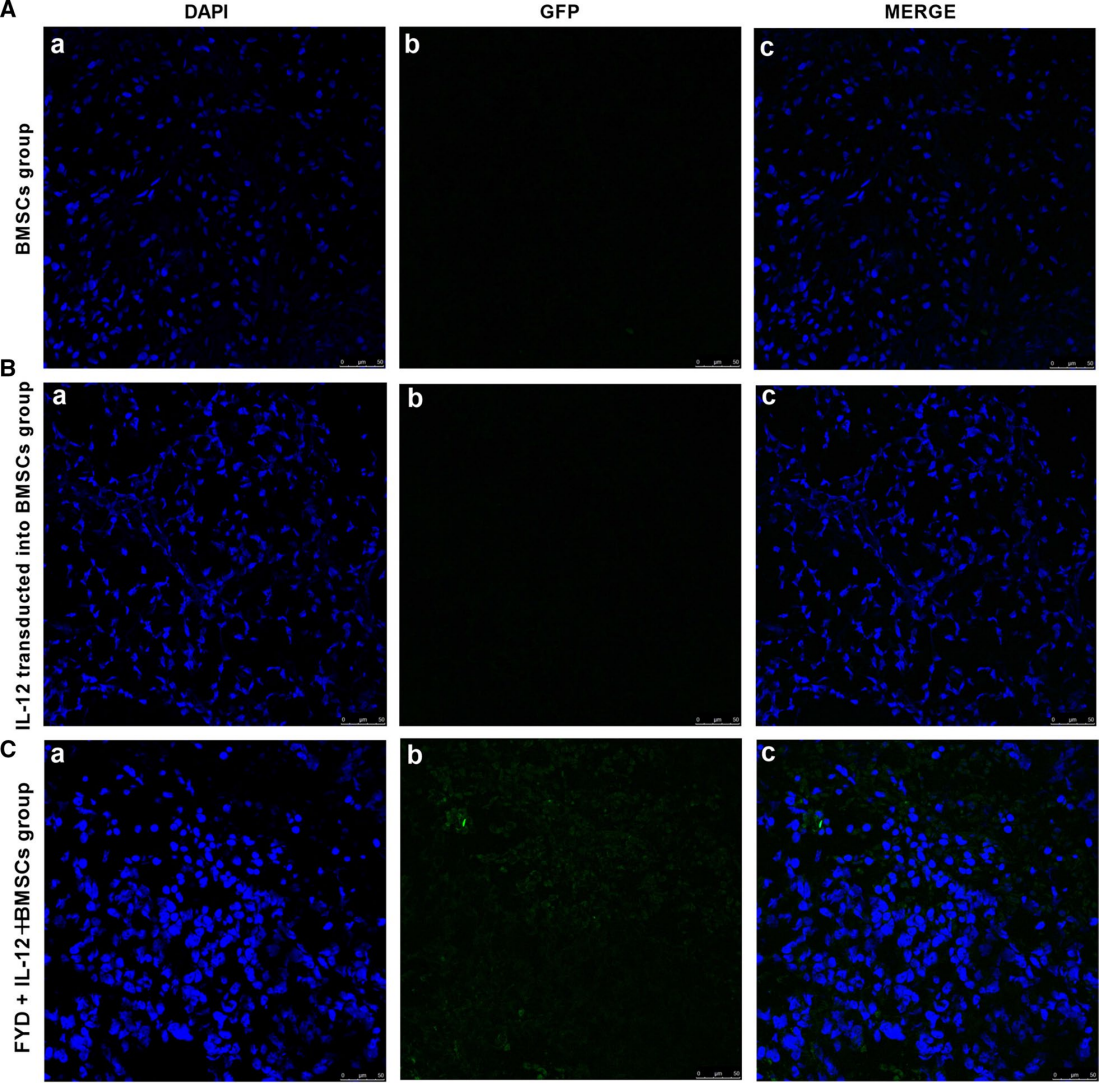
Fluorescent distribution characteristics of IL-12-transfected BMSCs and induction of apoptosis measured by DAPI staining in mice (×400). (Aa), (Ba) and (Ca) indicate DAPI staining results in the BMSC, IL-12 + BMSC and FYD + IL-12 + BMSC groups. (Ab), (Bb) and (Cb) indicate the fluorescence data in the BMSC, IL-12 + BMSC and FYD + IL-12 + BMSC groups. (Ac), (Bc) and (Cc) indicate DAPI staining and fluorescence merged signals in the same field using confocal microscopy for the BMSC, IL-12 + BMSC and FYD + IL-12 + BMSC groups. Fluorescence was not observed in the tumor tissue of mice in the BMSC group, whereas it was evident in the tumor tissues from the IL-12 + BMSC and FYD + IL-12 + BMSC groups, indicating that the IL-12 gene was successfully transfected into BMSCs (**a**–**c**). The induction of apoptosis was measured by DAPI staining. Apoptosis was more prevalent in the IL-12 + BMSC and FYD + IL-12 + BMSC groups compared with that of the BMSC group. The FYD + IL-12 + BMSC group exhibited a higher number of apoptotic cells showing blue fluorescence staining of cells compared with that of the IL-12 + BMSC group. The IL-12 + BMSC group exhibited a higher proportion of apoptotic cells as determined by blue fluorescence staining compared with the BMSC group (**a**–**c**). The results indicated that the IL-12 gene was distributed in tumor tissues, suggesting a homing effect of BMSCs with regard to IL-12. BMSCs, bone marrow mesenchymal stem cells; DAPI, 4′,6-diamidino-2-phenylindole; FYD, FuzhengYiliu decoction IL-12 + BMSC, IL12-lentiviral-transfected BMSC; BMSC group, empty lentiviral vector-transfected BMSC group; FYD + IL-12 + BMSC group, FYD treatment in IL-12 lentiviral-transfected BMSC group

Following immunofluorescence staining, confocal microscopy was used to detect fluorescence intensity. The fluorescence signals were not observed in the tumor tissues of the mice in the model group, whereas they were evident in the tumor tissues of the BMSC group. Weaker fluorescence signals were emitted by GFP in the latter group, whereas the IL-12 + BMSC group exhibited a stronger GFP-emitted fluorescence signal, indicating that the IL-12 gene was successfully transfected into BMSCs. The results indicated that the IL-12 gene was distributed in tumor tissues, revealing a homing effect of BMSCs with regard to IL-12 (Fig. 3a–c).

### Estimation of the number of apoptotic cells as determined by DAPI and TUNEL staining

DAPI staining indicated that apoptosis was more prevalent in the IL-12 + BMSC and FYD + IL-12 + BMSC groups compared with that noted in the BMSC group. The FYD + IL-12 + BMSC group exhibited a higher number of apoptotic cells as demonstrated by blue fluorescence staining compared with that of the IL-12 + BMSC group. The IL-12 + BMSC group exhibited a higher number of apoptotic cells as determined by blue fluorescence staining compared with that of the BMSC group (Fig. 3a and b).

**Fig. 4:**
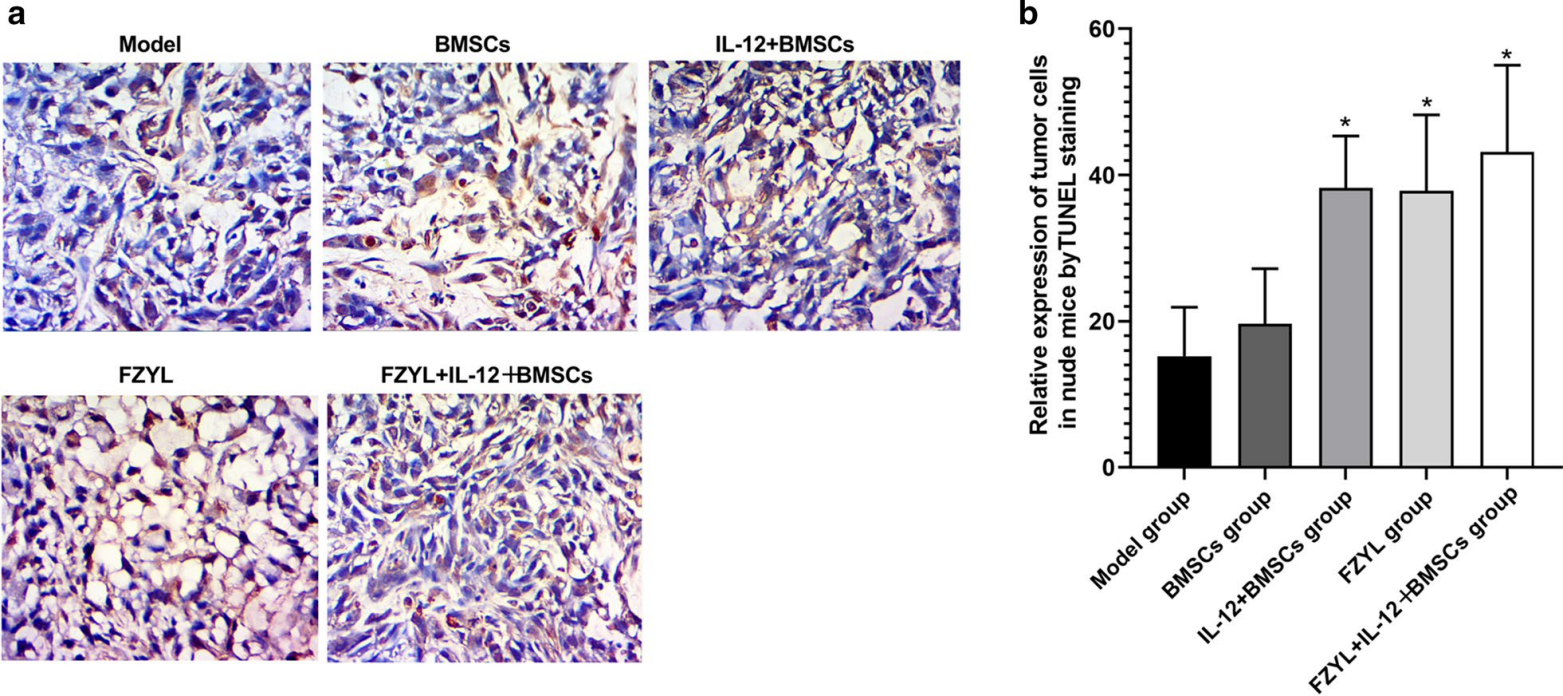
Evaluation of tumor cell apoptosis in nude mice by TUNEL staining (×400). The induction of apoptosis was measured by the TdT-mediated dUPT nick end labeling staining method. Apoptosis was more prevalent in the IL-12 + BMSC, FYD and FYD + IL-12 + BMSC groups compared with the corresponding percentages noted in the model group. The FYD + IL-12 + BMSC group exhibited a higher proportion of apoptotic cells demonstrating dark-brown nuclear staining, which also exhibited a withered appearance; for comparison, the nuclei of the non-apoptotic cells were counterstained blue with hematoxylin, as shown in **a** and **b**. n = 5, The relative number of the apoptosis-positive cells was higher in the IL-12 + BMSC, FYD, and FYD + IL-12 + BMSC groups compared with that of the model group (P < 0.05). TUNEL, TdT-mediated dUPT nick end labeling; BMSCs, bone marrow mesenchymal stem cells; IL-12 + BMSC, IL12-lentiviral-transfected BMSC; BMSC group, empty lentiviral vector-transfected BMSC group; FYD, FuzhengYiliu decoction; FYD + IL-12 + BMSC group, FYD treatment in IL-12 lentiviral-transfected BMSC group

TUNEL staining results indicated that apoptosis was more prevalent in the IL-12 + BMSC group, FYD group and FYD + IL-12 + BMSC group compared with that noted in the model group. The FYD + IL-12 + BMSC group exhibited a higher number of apoptotic cells demonstrating a dark-brown nuclear staining. For comparison, the nuclei of the non-apoptotic cells were counterstained blue with hematoxylin (Fig. 4a and b). The relative expression levels of the apoptotic cells was higher in the IL-12 + BMSC, FYD, and FYD + IL-12 + BMSC groups compared with those of the model group (P < 0.05).

### Serum IL-12 and IFN-γ levels

**Fig. 5:**
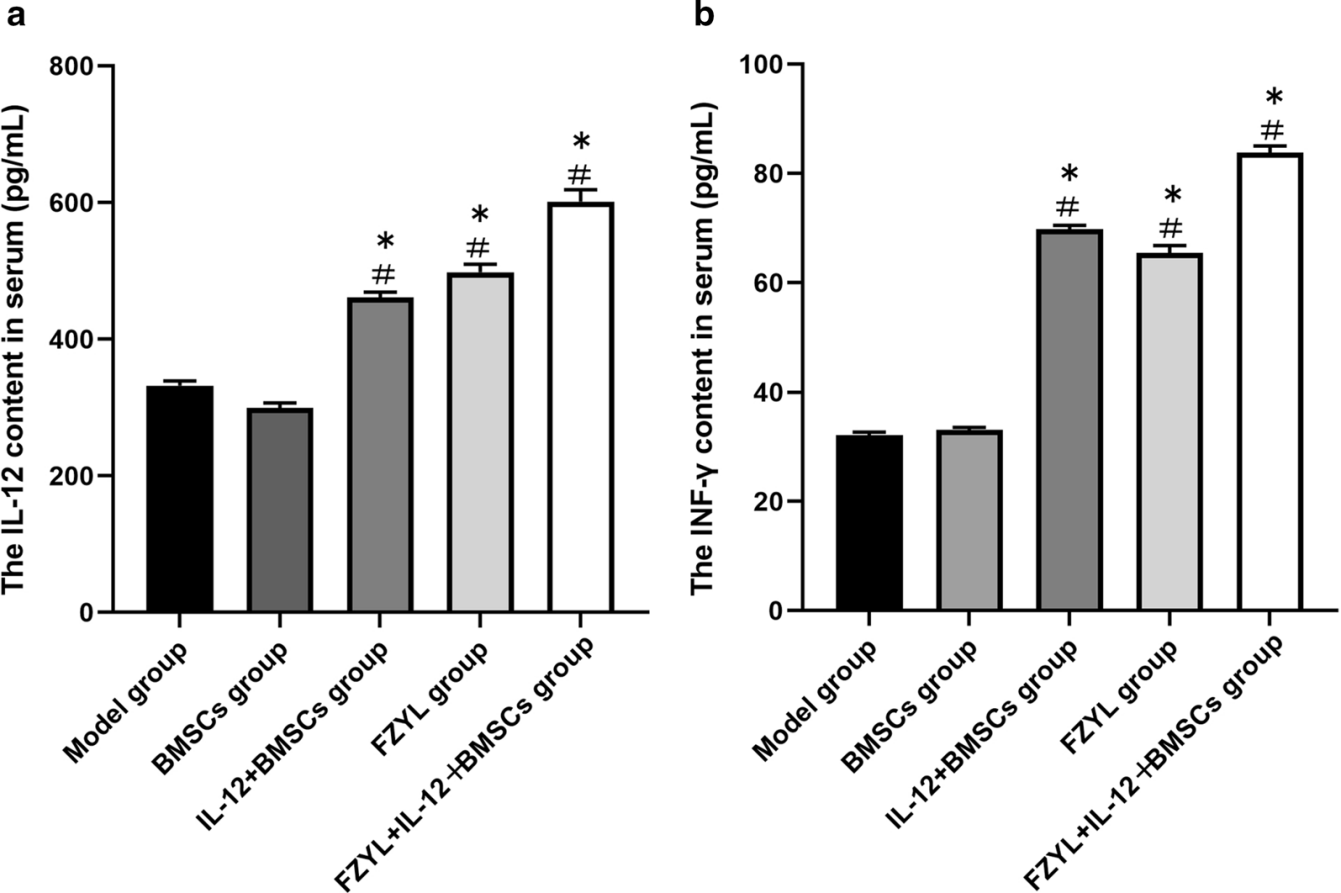
Detection of serum IL-12 and IFN -γ levels. The levels of IL-12 differed significantly among the groups (P < 0.05). The highest IL-12 levels were noted in the FYD + IL-12 + BMSC group (P < 0.05). In particular, significant differences were noted between the IL-12 + BMSC and FYD groups and between the IL-12 + BMSC and FYD + IL-12 + BMSC groups (P < 0.05). In addition, significant differences were noted with regard to IFN-γ levels (P < 0.05) among the groups, with the highest levels observed in the FYD + IL-12 + BMSC group (P < 0.05), as shown in **a**, **b**. n = 5, ^#^P < 0.05: Compared with the model group, ^*^P < 0.05; compared with the model group. One-way analysis of variance was performed (IL-12: F = 1107.69, P = 0.00; IFN-γ: F = 5456.68, P = 0.00). The q-test was used to compare the two groups and significant differences were noted between the two groups (P < 0.05). BMSCs, bone marrow mesenchymal stem cells; IL-12 + BMSC, IL12-lentiviral-transfected BMSC; FYD, FuzhengYiliu decoction; FYD + IL-12 + BMSC group, FYD treatment in IL-12 lentiviral-transfected BMSC group

The expression levels of IL-12 differed significantly among the groups (P < 0.05), among which the FYD + IL-12 + BMSC group exhibited the highest level of IL-12 (P < 0.05). In particular, significant differences were noted between the IL-12 + BMSC and FYD groups and between the IL-12 + BMSC and FYD + IL-12 + BMSC groups (P < 0.05). In addition, significant differences were noted in the levels of IFN-γ (P < 0.05) among the groups, with the highest levels observed in the FYD + IL-12 + BMSC group (P < 0.05) (Fig. 5a and b).

### Bax and Bcl-2 expression levels in tumor tissues

**Fig. 6:**
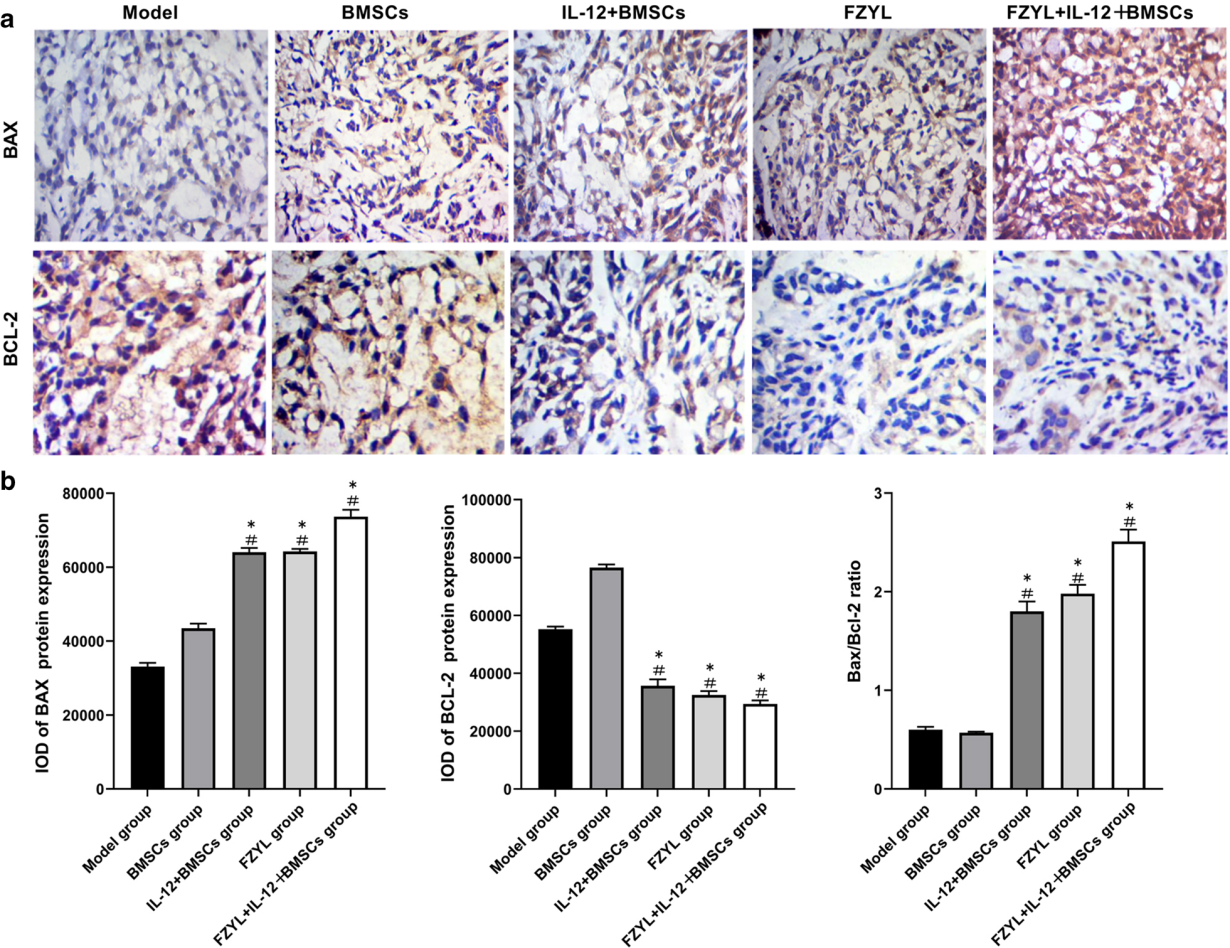
Bax and Bcl-2 expression in tumor tissues**.** Immunohistochemical staining indicated that Bax and Bcl-2 were mainly located in the cytoplasm, as evidenced by a clear brown-yellow diffuse distribution. **a** Some positive cells also exhibited brown particles in the cytoplasm or nuclear membrane (**a** and **b**). The expression levels of Bax were significantly higher in the IL-12 + BMSC, FYD, and FYD + IL-12 + BMSC groups following intervention compared with those of the model and BMSC groups (P < 0.05), whereas Bcl-2 expression was downregulated in the IL-12 + BMSC, FYD, and FYD + IL-12 + BMSC groups compared with that of the model and BMSC groups (P < 0.05). n = 3, ^#^P < 0.05: Compared with the model group, ^*^P < 0.05: Compared with the model group. BMSCs, bone marrow mesenchymal stem cells; IL-12 + BMSC, IL12-lentiviral-transfected BMSC; BMSC group, empty lentiviral vector-transfected BMSC group; FYD, FuzhengYiliu decoction; FYD + IL-12 + BMSC group, FYD treatment in IL-12 lentiviral-transfected BMSC group

Immunohistochemical staining indicated that Bax and Bcl-2 were mainly located in the cytoplasm, as demonstrated by a clear brown-yellow diffuse distribution. Some positive cells contained brown particles in the cytoplasm or nuclear membrane (Fig. 6a). Bax expression levels were significantly higher in the IL-12 + BMSC, FYD and FYD + IL-12 + BMSC groups following intervention compared with those noted in the model and BMSC groups (P < 0.05). Bcl-2 expression levels were downregulated in the IL-12 + BMSC, FYD and FYD + IL-12 + BMSC groups compared with those noted in the model and BMSC groups (P < 0.05) (Fig. 6a and b).

### Tumor volume and body weight changes in tumor-bearing mice

**Fig. 7:**
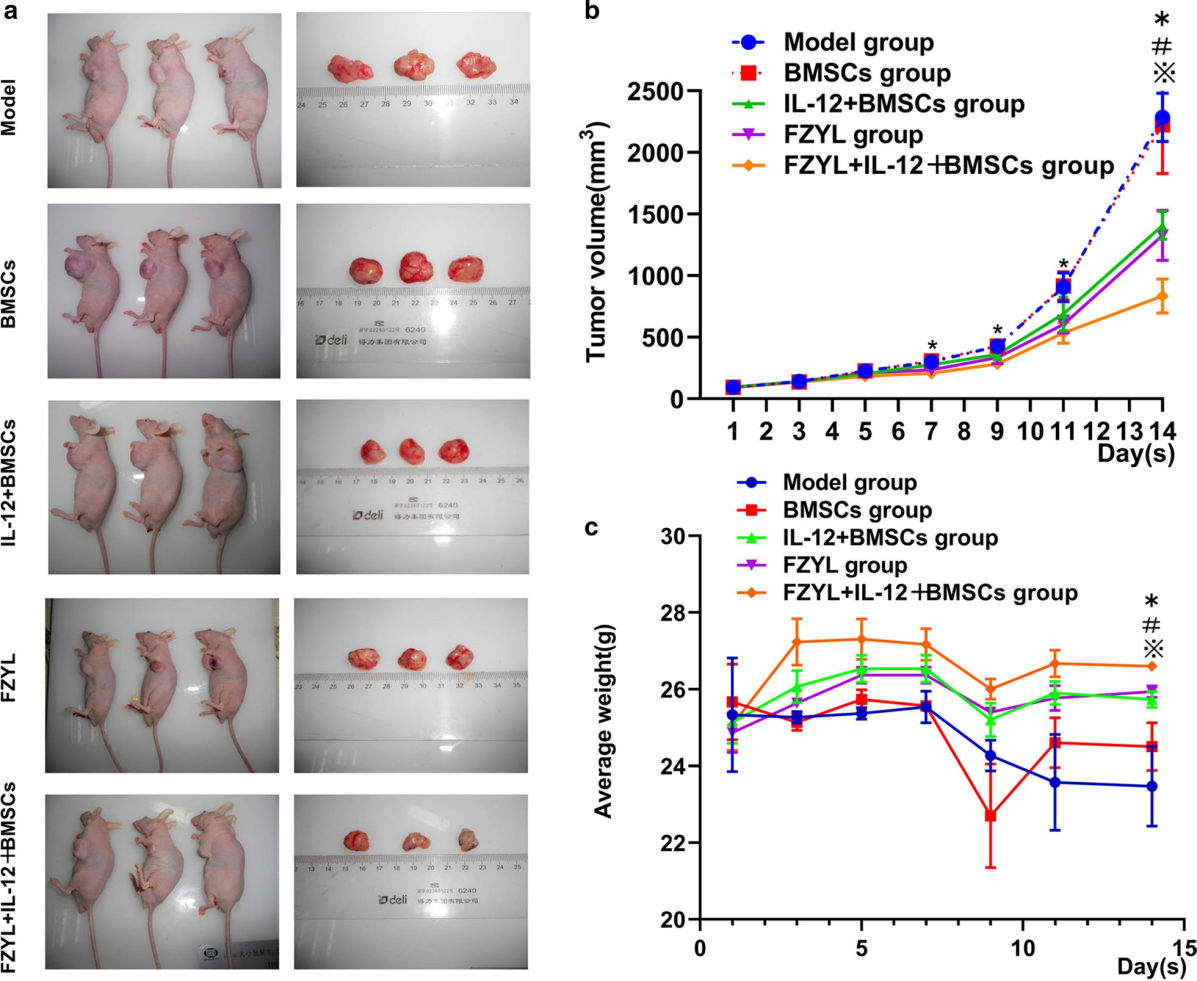
Tumor volume and body weight changes in tumor-bearing mice. The tumor volume and body weight of tumor-bearing mice were measured every other day during the 14-day period of intragastric FYD administration. The mean tumor volume change of each group was recorded and calculated daily. **a** These figures showed the tumor-bearing mice and tumor. **b** A growth curve was drawn based on the tumor volume change in each group, which revealed the trend in tumor volume growth in each experimental group. The tumor volume growth in the FYD + IL-12 + BMSC group was significantly lower than that of the model and BMSC groups. For the comparison of tumor volume, the sphericity test *P* = 0.00 < 0.01, which did not conform to the sphericity, and the Greenhouse–Geisser model gives *F* = 482.8, *P* = 0.00, indicating that the main effect difference was statistically significant at different time points. The comparison of Tukey’s test between the two groups found that the difference between any two groups was statistically significant (*P* = 0.00); and the interaction test time **╳** group found *F* = 16.79, *P* = 0.00, indicating there is interaction with the group. In addition, the tests of between-subjects effects test found statistically significant differences in the main effects between the different groups (*F* = 28.22, *P* = 0.00). n = 3, ^*^P < 0.05: On the 7th, 9th, 11th and 14th days, IL-12 + BMSCs group, FYD group, FYD + IL-12 + BMSCs group *vs* Model group, only except on the 9th and 11th day, IL-12 + BMSCs group *vs* Model group. ^#^P < 0.05, On 14th day, with the exception of the FYD group, all other groups *vs* IL-12 + BMSCs group. ^※^P < 0.05: On 14th day, all other groups *vs* the FYD + IL-12 + BMSCs group. **c** The body weight of the FYD + IL-12 + BMSC group was significantly lower than that of all other groups. For the comparison of body weight changes in tumor-bearing mice, the sphericity test which does not conform to the sphericity (*P* = 0.00 < 0.01). There were statistically different in the main effect on time points with Greenhouse–Geisser model *F* = 13.73, *P* = 0.00. Interaction test time **╳** group found that there was interaction between time and group (*F* = 3.053, *P* = 0.02). In addition, there was a statistically significant difference in the main effect between the different groups on the 14th day by the tests of Between-Subjects Effects using Tukey’s test method (*F* = 75.779, *P* = 0.00). n = 3, ^*^P < 0.05: On the 14th day, IL-12 + BMSCs group, FYD group, FYD + IL-12 + BMSCs group *vs* Model group, ^#^P < 0.05: On the 14^th^ day: all other groups *vs* all other groups *vs* IL-12 + BMSCs group, ^※^P < 0.05: All other groups *vs* the FYD + IL-12 + BMSCs group. Note: BMSCs, bone marrow mesenchymal stem cells; IL-12 + BMSC, IL12-lentiviral-transfected BMSC; BMSC group, empty lentiviral vector-transfected BMSC group; FYD, FuzhengYiliu decoction; FYD + IL-12 + BMSC group, FYD treatment in IL-12 lentiviral-transfected BMSC group

The tumor volume and body weight of tumor-bearing mice were measured every other day during the 14-day period of intragastric FYD administration. The mean tumor volume change of each group was calculated daily. The diameter of a single tumor varies from 8.12 to 12.38 mm. A growth curve was drawn according to the tumor volume change in each group, which revealed the trend in tumor volume growth in each experimental group. The tumor volume growth in the IL-12 + BMSCs group, FYD group, FYD + IL-12 + BMSCs groups was significantly lower than that of the model and BMSC groups on the 7th, 9th, 11th and 14th days, only except IL-12 + BMSCs group *vs* Model group on the 9th and 11th days, and the tumor volume growth in the FYD + IL-12 + BMSCs group was significantly lower than that of all other groups on 14^th^ day and there was no significant difference between FYD + IL-12 + BMSCs group and FYD group on 14th day (Fig. 7b, P < 0.05). A growth curve was drawn according to the body weight changes in tumor-bearing mice in each group, which revealed the trend in the body weight changes in each experimental group. The body weight in the FYD + IL-12 + BMSC group was significantly lower than that of the model group on 14th day (Fig. 7C, P < 0.05).

## Discussion

Glioma is an invasive and infiltrative type of tumor, and is the most common type of primary brain cancer. Treatment of glioma is complicated by glioma stem cells that induce immune tolerance in the microenvironment [30]. Thus, the complex features of malignant glioma contribute to a poor prognosis [31], with both high morbidity and mortality [32].

BMSCs have been used as drug/gene targeted carriers to treat gliomas, but the results largely depend on their specific homing ability [33, 34]. In this study, cell morphology and cell surface immune marker expression were consistent with those reported in the literature [3, 8, 11, 33, 34]. The IL-12 gene was successfully transfected into BMSCs, BMSCs with the IL-12 gene had a strong homing effect in tumor tissues, and the reliability of BMSCs as a tumor bio therapeutic vector was verified. Cell-based antitumor targeted therapies must rely on the ability of BMSCs to activate localized tumor tissues and their intrinsic ability to influence target cells; however, the safety of BMSCs as the preferred vector for biotherapy remains unclear [3].

Considerable progress has been made in the study of BMSC to treat glioma. However, no study has investigated the treatment of malignant glioma using BMSCs because of the difficulty in determining the best treatment time post-BMSC implantation and the assessment of long-term outcomes post-treatment. Regardless, this targeted therapy remains promising as a treatment for most solid tumors, including glioma [35, 36]. In particular, BMSC-based therapy is an effective antitumor treatment because of its tumor-tropic homing and migration properties^29^. In the present study, when the IL-12 gene was transfected into BMSCs and IL-12 was stably expressed in serum (*P* < 0.05), a significant inhibitory effect on tumor growth was observed. Specifically, there was a significant difference between the model group and the IL-12 + BMSC, FYD, and FYD + IL-12 + BMSC groups on days 9, 11, and 14 in tumor-bearing mice (*P* < 0.05). The model group also exhibited significant tumor-induced weight loss (*P* < 0.05).

Studies have shown that BMSCs have a similar self-renewal ability as cancer cells, and possess the ability to transform spontaneously in vitro. It has also been suggested that tumors may transform from highly invasive mesenchymal stem cells [37], especially in the tumor microenvironment [38]. In addition, Japanese scientists confirmed that there was still a significant difference in the expression of proliferating genes between BMSCs and cancer cells [39]. Thus, BMSC use remains limited in clinical application because of intrinsic uncertainties in their outcome and possible tumorigenicity.

Clinical use of TCM to treat tumors has many advantages [40, 41]. For example, Fu Zheng and exorcism therapies are the main TCM treatments for tumors [42, 43], and Fu Zheng treatment has been shown to improve immune function [26, 44, 45]. Our previous study showed that serum containing FYD could reverse the tumorigenicity of BMSCs in the glioma microenvironment by inhibiting the telomerase activity of BMSCs, increasing the expression of p53, and inducing apoptosis in vivo [27, 46]. Furthermore, the present study confirmed that the TCM FYD could effectively inhibit the potential tumorigenicity of BMSCs in mice, and that BMSCs expressing the IL-12 gene had a targeted antitumor effect based on TUNEL staining, tumor volume and body weight changes, serum IL-12 levels, and Bax/Bcl-2 expression. Among all treatment groups (including the FYD and IL-12 + BMSC groups) in the present study, the combined FYD + IL-12 + BMSC group showed a significantly better outcome.

Previous reports have shown that the inhibitory effect of BMSCs on tumors might depend on the involvement of immune mechanisms [11, 47]. IL-12 has emerged as a key molecule in modulating both innate and adaptive immune responses and mediating antitumor activity [48]. For instance, IL-12 can cause CD4^+^ CD8^+^ cells to infiltrate and secrete various cytokines (e.g., IFN-γ) into the tumor microenvironment, which precipitates cascade reactions via other cytokines to elicit an antitumor effect [48, 49]. As such, IL-12 is increasingly favored as a candidate for immune-mediated gene therapy, which inspired the use of IL-12 as the target gene in the present research.

After routine therapy to remove a large proportion of tumor tissue from solid tumors, immunotherapy can be used to remove residual cells and enhance the therapeutic effect. However, surgery, chemotherapy, radiotherapy, and other treatments, can weaken the immune systems of cancer patients, which is an important consideration in the final treatment of tumors [50]. Because the model nude mice in the present study exhibit T cell immunodeficiency, the mice were very similar to the clinical environment after tumor transplantation. IL-12 stimulates the proliferation of natural killer (NK) cells and macrophages to enhance their natural killing function, and NK cells also become sensitive to NK killing, resulting in reduced tumor progression. IL-12 exerts an antitumor effect by helping to inhibit tumor angiogenesis, reducing microvascular density, and inducing apoptosis through the production of IFN-γ [49]. Our results showed that serum IL-12 and IFN-γ levels were significantly higher in the IL-12 + BMSC and FYD + IL-12 + BMSC groups than in the other groups, as detected by enzyme-linked immunosorbent assay (ELISA) (*P* < 0.05).

A high Bax/Bcl-2 ratio can cause a reduction in mitochondrial membrane potential, which is an early intracellular signal that leads to apoptosis [51]. In the present study, the cellular expressions of the Bax and Bcl-2 apoptotic markers in the intervention groups were significantly higher than in the model group. In addition, among all treatment groups, the FYD + IL-12 + BMSC group showed the highest serum IL-12 and IFN-γ levels and Bax and Bcl-2 expressions in tumor cells (*P* < 0.05).

In summary, the present study demonstrated the successful application of the combined treatment of FYD and IL-12-transfected BMSCs. However, more investigations are required to determine whether IL-12 activates antigen-presenting cells (i.e. NK cells and macrophages) and whether FYD promotes non-specific immunity in tumor-bearing nude mice, and the experiments of functional and molecular mechanisms in vitro associated with tumor phenotype, such as, proliferation, apoptosis, metastasis and others are needed to be detected in U251-MG cells to explore relevant mechanisms.

## Data Availability

All data and materials generated and analyzed during the present study are available from the corresponding author on reasonable request.

## Abbreviations

FYD: Fuzheng Yiliu decoction
BMSCs: Bone marrow mesenchymal stem cells
TLRs: Toll-like receptors
TCM: Traditional Chinese Medicine
PBS: Phosphate-buffered saline

## References

[1] Chen J, McKay RM, Parada LF (2012). Malignant glioma: lessons from genomics, mouse models, and stem cells. Cell.

[2] Pittenger MF, Mackay AM, Beck SC, Jaiswal RK, Douglas R, Mosca JD, Moorman MA, Simonetti DW, Craig S, Marshak DR (1999). Multilineage potential of adult human mesenchymal stem cells. Science.

[3] Motaln H, Schichor C, Lah TT (2010). Human mesenchymal stem cells and their use in cell-based therapies. Cancer.

[4] Kim SW, Lee YK, Hong JH, Park JY, Choi YA, Lee DU, Choi J, Sym SJ, Kim SH, Khang D (2018). Mutual destruction of deep lung tumor tissues by nanodrug-conjugated stealth mesenchymal stem cells. AdvSci.

[5] Zhang S, Liu Y, Derakhshanfar S, He W, Huang Q, Dong S, Rao J, Luo GX, Zhong W, Liao W (2018). Polymer self-assembled BMSCs with cancer tropism and programmed homing. AdvHealthc Mater.

[6] Du J, Zhou L, Chen X, Yan S, Ke M, Lu X, Wang Z, Yu W, Xiang AP (2012). IFN-gamma-primed human bone marrow mesenchymal stem cells induce tumor cell apoptosis in vitro via tumor necrosis factor-related apoptosis-inducing ligand. Int J Biochem Cell Biol.

[7] Romieu-Mourez R, Francois M, Boivin MN, Bouchentouf M, Spaner DE, Galipeau J (2009). Cytokine modulation of TLR expression and activation in mesenchymal stromal cells leads to a proinflammatory phenotype. J Immunol.

[8] Zhang TY, Huang B, Yuan ZY, Hu YL, Tabata Y, Gao JQ (2014). Gene recombinant bone marrow mesenchymal stem cells as a tumor-targeted suicide gene delivery vehicle in pulmonary metastasis therapy using non-viral transfection. Nanomedicine.

[9] Wei Z, Chen N, Guo H, Wang X, Xu F, Ren Q, Lu S, Liu B, Zhang L, Zhao H (2009). Bone marrow mesenchymal stem cells from leukemia patients inhibit growth and apoptosis in serum-deprived K562 cells. J ExpClin Cancer Res.

[10] Mi F, Gong L (2017). Biosci Rep.

[11] Zhang L, Su XS, Ye JS, Wang YY, Guan Z, Yin YF (2015). Bone marrow mesenchymal stem cells suppress metastatic tumor development in mouse by modulating immune system. Stem Cell Res Ther.

[12] Mognetti B, La Montagna G, Perrelli MG, Pagliaro P, Penna C (2013). Bone marrow mesenchymal stem cells increase motility of prostate cancer cells via production of stromal cell-derived factor-1alpha. J Cell Mol Med.

[13] Shirjang S, Mansoori B, Solali S, Hagh MF, Shamsasenjan K (2017). Toll-like receptors as a key regulator of mesenchymal stem cell function: an up-to-date review. Cell Immunol.

[14] Romieu-Mourez R, Francois M, Boivin MN, Stagg J, Galipeau J (2007). Regulation of MHC class II expression and antigen processing in murine and human mesenchymal stromal cells by IFN-gamma, TGF-beta, and cell density. J Immunol.

[15] Duan X, Guan H, Cao Y, Kleinerman ES (2009). Murine bone marrow-derived mesenchymal stem cells as vehicles for interleukin-12 gene delivery into Ewing sarcoma tumors. Cancer.

[16] Dahl JA, Duggal S, Coulston N, Millar D, Melki J, Shahdadfar A, Brinchmann JE, Collas P (2008). Genetic and epigenetic instability of human bone marrow mesenchymal stem cells expanded in autologous serum or fetal bovine serum. Int J Dev Biol.

[17] El-Badawy A, Ghoneim MA, Gabr MM, Salah RA, Mohamed IK, Amer M, El-Badri N (2017). Cancer cell-soluble factors reprogram mesenchymal stromal cells to slow cycling, chemoresistant cells with a more stem-like state. Stem Cell Res Ther.

[18] Gjerstorff M, Burns JS, Nielsen O, Kassem M, Ditzel H (2009). Epigenetic modulation of cancer-germline antigen gene expression in tumorigenic human mesenchymal stem cells: implications for cancer therapy. Am J Pathol.

[19] Qi F, Li A, Inagaki Y, Gao J, Li J, Kokudo N, Li XK, Tang W (2010). Chinese herbal medicines as adjuvant treatment during chemo- or radio-therapy for cancer. Biosci Trends.

[20] Liu XP, Ming HX, Li PQ (2016). Intervention effect of pinelliae decoction for purging stomach-fire on malignant transformation of bone marrow mesenchymal stem cells in the gastric cancer microenvironment. Am J Transl Res.

[21] Ma YX, Liu LQ, Qin LM, Zuo GM, Wang Y (2013). Xuefuzhuyu decoction containing serum in vitro induced expressions of desmin and alpha-actin: an experimental research. Chin J IntegrTradit West Med..

[22] Ke B, Shi L, Xu Z, Wu G, Gong Y, Zhu L, Wang Y, Ouyang H, Wang X (2018). Flavored GuiluErxian decoction inhibits the injury of human bone marrow mesenchymal stem cells induced by cisplatin. Cell MolBiol.

[23] Yang P, Chen A, Qin Y, Yin J, Cai X, Fan YJ, Li L, Huang HY (2019). Buyanghuanwu decoction combined with BMSCs transplantation promotes recovery after spinal cord injury by rescuing axotomized red nucleus neurons. J Ethnopharmacol.

[24] Wang XX, Zhao JX, Chen R (2007). Effect of FuzhengYiliu granule contained-serum from tumor bearing mice on apoptotic rate, free radicals content and mitochondrial membrane potential of hepatoma cell lines H22 in vitro. Chin J IntegrTradit West Med.

[25] Zhao JX (2006). Effects of FuzhengYiliu Granules on apoptotic rate and mitochondrial membrane potential of hepatocellular carcinoma cell line H22 from mice. Chin J IntegrTradit West Med.

[26] Zhao JX, Li XF, Wang XX (2007). Effects of body-resistance strengthening and tumor-suppressing granules on immune adhesion function of red blood cells and expession of metastasis protein CD44 in tumor cells of patients with esophageal carcinoma. World J Gastroenterol.

[27] Zeng L, Ma Z, Wu J, Wang Z, Zhou S, Wang X (2016). The effect of FuzhengYiliu decoction containing serum on proliferation, cycle and apoptosis of bone marrow mesenchymal stem cells in glioma environment. J Lanzhou Univ..

[28] IoLA R (2011). Guide for the care and use of laboratory animals. Publication.

[29] Cima G (2013). AVMA Guidelines for the Euthanasia of Animal 2013 Edition. J Am Vet Med Assoc.

[30] Xi G, Best B, Mania-Farnell B, James CD, Tomita T (2017). Therapeutic potential for bone morphogenetic protein 4 in human malignant glioma. Neoplasia.

[31] Qing Z, Wei X, Dong-ye Y, Bing-zhou X, Wan-wan W, Ahmed A, Nan-xiang X, Xiao-bing J, Hong-yang Z, Peng F (2017). Current status and potential challenges of mesenchymal stem cell-based therapy for malignant gliomas. Stem Cell Research & Therapy.

[32] Dunn-Pirio AM, Vlahovic G (2017). Immunotherapy approaches in the treatment of malignant brain tumors. Cancer.

[33] Xiaoling W, Jian-Qing G, Xumei O, Junbo W, Xiaoyi S, Yuanyuan L (2018). Mesenchymal stem cells loaded with paclitaxel–poly(lactic-co-glycolic acid) nanoparticles for glioma-targeting therapy. International Journal of Nanomedicine.

[34] Shi S, Min Z, Rui G, Ying M, Biao L: Bone Marrow Derived Mesenchymal Stem Cell-Mediated Dual-gene Therapy for Glioblastoma. *Human Gene Therapy*:hum.2018.2092-. 10.1089/hum.2018.092 PMC6909702 29993289

[35] Mooney R, Hammad M, Batalla-Covello J, Abdul Majid A, Aboody KS (2018). Concise review: neural stem cell-mediated targeted cancer therapies. Stem Cells Transl Med.

[36] Stepanenko AA, Chekhonin VP: Recent Advances in Oncolytic Virotherapy and Immunotherapy for Glioblastoma: A Glimmer of Hope in the Search for an Effective Therapy? *Cancers (Basel)* 2018, 10. 10.3390/cancers10120492 PMC6316815 30563098

[37] Matushansky I, Hernando E, Socci ND, Mills JE, Cordon-Cardo C (2007). Derivation of sarcomas from mesenchymal stem cells via inactivation of the Wnt pathway. J ClinInvestig.

[38] Zhao Y, Chen J, Dai X, Cai H, Ji X, Sheng Y, Liu H, Yang L, Chen Y, Xi D (2017). Human glioma stem-like cells induce malignant transformation of bone marrow mesenchymal stem cells by activating TERT expression. Oncotarget.

[39] Sawada R, Matsuoka A, Matsuda Y, Tsuchiya T (2008). Change in characteristics of human mesenchymal stem cells during the in vitro culture. YakugakuZasshi.

[40] Zheng X, Wu F, Lin X, Shen L, Feng Y (2018). Developments in drug delivery of bioactive alkaloids derived from traditional Chinese medicine. Drug Deliv.

[41] Pu WL, Sun LK, Gao XM, Ruegg C, Cuendet M, Hottiger MO, Zhou K, Miao L, Zhang YS, Gebauer M (2017). Targeting tumor-associated macrophages by anti-tumor Chinese materiamedica. Chin J Integr Med.

[42] Fu'er L, Guangying H (2003). Investigation on therapeutic mechanisms of three principles of traditional Chinese medicine for treating malignant tumors. Chin J Integr Med.

[43] Kai J, Yang D: Clinical study of the combined treatment with Chinese traditional medicine and interventional chemotherapy on malignant tumor. Modern J Integr Tradit Chin West Med 2009.

[44] Chen XZ, Cao ZY, Liao LM, Liu ZZ, Jian D (2013). Application of serum pharmacology in evaluating the antitumor effect of FuzhengYiliu Decoction from Chinese medicine. Chin J Integr Med.

[45] Wei F, xin LQ, shu CB, bei ZB, yan YG, long CC, ming LW, Lin Y, qiu ZB, jun YX: Study on Tumor Suppressing Effect of Fuzheng Yiliu Decoction on Prostate Cancer Bearing Nude Mice and Expression of Serum IL-2. *Guiding Journal of Traditional Chinese Medicine and Pharmacy* 2018.

[46] Jianjun W, Long Z, Yongqi L, Jia Y, Zhanjun M, Zhuanling W, Shuning Z, Xuexi W: Effects of Fuzheng Yiliu Fang on Telomerase Activity and P53 Expression in BMSCs in C6 Micro-environment. *Chinese Journal of Traditional Medical Science and Technology* 2017.

[47] Chao S, Jia T, Mendez-Ferrer S, Hohl TM, Serbina NV, Lipuma L, Leiner I, Li MO, Frenette PS, Pamer EG (2011). Bone marrow mesenchymal stem and progenitor cells induce monocyte emigration in response to circulating toll-like receptor ligands. Immunity..

[48] Kim H, Gao W, Ho M (2013). Novel immunocytokine IL12-SS1 (Fv) inhibits mesothelioma tumor growth in nude mice. Plos One.

[49] Yang SX, Wei W-S, Ouyan Q-W, Jiang Q-H, Zou Y-F, Qu W, Tu J-H, Zhou Z-B, Ding H-L, Xie C-W (2016). Interleukin-12 activated CD8+ T cells induces apoptosis in breast cancer cells and reduces tumor growth. Biomed Pharmacother..

[50] Zuo S, Zhang X, Wang L (2019). A RNA sequencing-based six-gene signature for survival prediction in patients with glioblastoma. Sci Rep.

[51] Skala E, Sitarek P, Toma M, Szemraj J, Radek M, Nieborowska-Skorska M, Skorski T, Wysokinska H, Sliwinski T (2016). Inhibition of human glioma cell proliferation by altered Bax/Bcl-2-p53 expression and apoptosis induction by *Rhaponticum carthamoides* extracts from transformed and normal roots. J Pharm Pharmacol.

